# Nonalcoholic fatty liver disease in inflammatory arthritis: Relationship with cardiovascular risk

**DOI:** 10.3389/fimmu.2022.997270

**Published:** 2022-09-23

**Authors:** Nuria Barbarroja, Miriam Ruiz-Ponce, Laura Cuesta-López, Carlos Pérez-Sánchez, Chary López-Pedrera, Iván Arias-de la Rosa, Eduardo Collantes-Estévez

**Affiliations:** Rheumatology service and Medical and Surgical Sciences Department, Maimonides Biomedical Research Institute of Cordoba (IMIBIC)/University of Cordoba/Reina Sofia University Hospital, Cordoba, Spain

**Keywords:** rheumatoid arthritis, psoriatic arthritis, non-alcoholic fatty liver disease, methotrexate, cardiovascular risk, liver disease

## Abstract

Liver disease is one of the most important causes of morbidity and mortality worldwide whose prevalence is dramatically increasing. The first sign of hepatic damage is inflammation which could be accompanied by the accumulation of fat called non-alcoholic fatty liver disease (NAFLD), causing damage in the hepatocytes. This stage can progress to fibrosis where the accumulation of fibrotic tissue replaces healthy tissue reducing liver function. The next stage is cirrhosis, a late phase of fibrosis where a high percentage of liver tissue has been replaced by fibrotic tissue and liver functionality is substantially impaired. There is a close interplay of cardiovascular disease (CVD) and hepatic alterations, where different mechanisms mediating this relation between the liver and systemic vasculature have been described. In chronic inflammatory diseases such as rheumatoid arthritis (RA) and psoriatic arthritis (PsA), in which the CVD risk is high, hepatic alterations seem to be more prevalent compared to the general population and other rheumatic disorders. The pathogenic mechanisms involved in the development of this comorbidity are still unraveled, although chronic inflammation, autoimmunity, treatments, and metabolic deregulation seem to have an important role. In this review, we will discuss the involvement of liver disease in the cardiovascular risk associated with inflammatory arthritis, the pathogenic mechanisms, and the recognized factors involved. Likewise, monitoring of the liver disease risk in routine clinical practice through both, classical and novel techniques and indexes will be exposed. Finally, we will examine the latest controversies that have been raised about the effects of the current therapies used to control the inflammation in RA and PsA, in the liver damage of those patients, such as methotrexate, leflunomide or biologics.

## 1 Introduction to inflammatory arthritis

Inflammatory arthritis involves a group of diseases whose main characteristic is the inflammation of different joints often leading to the functional impairment. Inflammatory arthritis can comprise forms of monoarthritis (affecting only one joint) and polyarthritis (affecting four or more joints), being included on this latest group rheumatoid arthritis (RA) and psoriatic arthritis (PsA).

Rheumatoid arthritis is a chronic inflammatory autoimmune disease, characterized by systemic inflammation that firstly affects the lining of synovial joints producing persistent synovitis in symmetric joints, leading to progressive disability and premature death (1). RA is distinguished by the presence of autoantibodies such as rheumatoid factor (RF) and antibodies directed against citrullinated peptides (ACPAs) (2, 3). ACPAs are considered the autoantibodies specific for RA and they have been related to specific genetic association patterns (4), more aggressive phenotypes, and different responses to treatment (5).

PsA is a complex and a heterogeneous inflammatory arthropathy, characterized by the presence of cutaneous plaques of psoriasis generally associated with joint inflammation, either axial or peripheral, that can significantly impair the quality of life (6, 7). PsA may also present various extra-articular manifestations such as enthesitis, dactylitis, and uveitis (7, 8).

The pathophysiology of these inflammatory arthritis involves the participation of different immune and other cells, such as macrophages, T and B cells, neutrophils, antigen-presenting cells (APC), endothelial cells, osteoclasts, keratinocytes or synovial fibroblast, and different immune modulators such as cytokines. The pathogenic mechanisms can be in some way specific of each disease. Thus, the cytokines mainly implicated in the pathogenesis of RA are tumor necrosis factor (TNF)-α, interleukin (IL)-6, IL-1, and IL-17, being key mediators of cell migration and inflammation. Through complex pathways, they actively participate in the joint destruction (9). Regarding PsA, keratinocytes recruit inflammatory dendritic cells to release pro-inflammatory interleukins IL-12 and IL-23, which in turn activate T-cells to produce other pro-inflammatory cytokines, such as IL-17, IL-22, interferon (IFN)-gamma and TNF-α. All these interleukins, in addition to producing skin lesions, are also involved in the arthritic manifestation of the disease (10).

Both PsA and RA can be associated with a number of extra-articular manifestations or comorbidities, including cardiovascular disorders, gastrointestinal, kidney, and lung diseases, metabolic alterations (obesity, type 2 diabetes mellitus (T2DM), metabolic syndrome, dyslipidemia), infections, osteoporosis, tumors, and depression (11–13). In addition, the liver injury might be considered an extra-articular manifestation of these inflammatory arthritis, especially the development of non-alcoholic fatty liver disease (NAFLD) (14, 15). However, there is some controversy between the occurrence of liver pathology as an extra-articular manifestation or as a product of hepatotoxicity of treatments used to reduce the impact of the inflammation.

In this review, we will discuss the relationship between RA and PsA, the rheumatic diseases with the highest prevalence of cardiometabolic comorbidities, and liver disease, the pathogenic mechanisms and factors involved in this association. NAFLD is clinically silent, we will describe the non-invasive cost-effectiveness approaches which rheumatologists could use to monitor liver disease risk in clinical practice. We will also examine the effects of the current treatments used to control the inflammation in RA and PsA in the liver damage. For this purpose the selection process included the searching of original and review publications, in English language, using the databases PubMed, Web of Science and Scopus, where the terms “NAFLD”, “non-alcoholic fatty liver disease”, “MAFLD”, “metabolic-associated fatty liver disease” or and “cardiovascular disease” or “Rheumatoid arthritis” or “Psoriatic arthritis” were used. Publications from the past 11 years were selected, although some highly regarded older publications were not excluded.

## 2 Cardiovascular risk in inflammatory arthritis

Patients with inflammatory arthritis have a higher prevalence and incidence of cardiovascular disease (CVD) than the general population, which may account for up to 40% of the mortality rate. In this sense, we and others have shown the highest prevalence of CVD risk factors in PsA followed by RA, compared to the rest of the inflammatory arthritis (16–18).

This increased risk has been attributed not only to the elevated prevalence of traditional risk factors (arterial hypertension (ATH), obesity, T2DM and hyperlipidemia), but also as a result of chronic systemic inflammation (19, 20). Most stablish CVD risk calculators underestimate the CVD risk among inflammatory arthritis. Thus, in RA patients, the EULAR recommendations stablished that CVD risk scores should be adapted by a 1.5 multiplication factor (21). The presence of carotid plaques is considered a reliable predictor of CVD. A number of studies affirm that patients with inflammatory arthritis, especially RA and PsA, show an increase in the development of early atherosclerosis (22, 23). Increased carotid intima media thickness has been reported in IA patients, even in subjects without established CVD risk factors (24). Thus to better determine cardiovascular risk, a combination of CVD risk prediction and carotid intima-media thickness (CIMT) measurement has been proposed in spondyloarthritis to improve the identification of cardiovascular risk in these patients (16, 25).

In the development of CVD, the endothelial dysfunction, in both large vessels and small vessels of the microvasculature, is a factor that significantly contributes as it usually precedes and can predict the development of atherosclerosis. Microvascular endothelial dysfunction is present in RA patients, although it seems that there is not a clear relationship with chronic inflammation and disease activity (26). However, endothelial dysfunction of large vessels measured by Flow-mediated dilation (FMD), has been found in early RA and is associated with autoimmunity, disease activity and HLADRB1*4 shared epitope (27). Similar to RA, several studies have reported a decreased FMD in PsA patients compared to controls [reviewed in (27)].

It is recognized that RA and PsA are associated with alterations in lipid pattern. These alterations are derived of the effect of inflammatory responses and mainly translated into a deregulation in the levels of cholesterol, triglycerides, LDL and HDL that are directly involved in the development of atherosclerosis (28). In fact, high levels of cholesterol and triglycerides were associated with subclinical atherosclerosis in PsA patients (24, 29). It is noteworthy to mention the existence of the RA-associated lipid paradox in which on one hand there is an inverse association between cholesterol and CV risks and secondly, treatments aimed to reduce inflammation induce certain elevations in lipid levels (30, 31).

Both, RA and PsA are associated with a number of metabolic comorbidities including obesity, insulin resistance (IR) and T2DM (32, 33). In this regard, our group recently reported that the inflammatory activity observed in RA patients is responsible for alterations in glucose and lipid homeostasis. Specifically, we evaluated the metabolic profile of 100 RA patients and 50 healthy donors and performed studies on both a collagen-induced arthritis (CIA) mouse model and an adipocyte cell line treated with serum from RA patients. Our findings indicated that RA-related metabolic dysregulation is dependent on inflammation and identified adipose tissue inflammation as the main cause of IR and the molecular dysregulation of glucose and lipid homeostasis (32). Following this line, our group recently confirmed an increased incidence of metabolic disorders in patients with PsA. Thus, levels of molecules involved in cardiovascular disease and adipocytokines were altered in patients with PsA and correlate with disease activity and the presence of metabolic comorbidities, suggesting a role of adipose tissue dysfunction in the pathogenesis of PsA (33). Due to the elevated rates of metabolic abnormalities such as obesity, metabolic syndrome (MetS) or T2DM, and the presence of chronic inflammation, the development of NAFLD might be expected to be more increased in patients with IA.

Although chronic inflammation is a key feature of RA and PsA, the mechanisms that contribute to CVD risk in these IA might be different. Systemic inflammation could directly contribute to CVD risk in RA, while in PsA, adiposity is the main responsible in conferring a metabolic phenotype that, in turn, contributes to CVD risk (34).

Thus, appropriate management strategies that consider the factors that involved in the increased CVD risk are critical. In the case of RA, these strategies could be aimed to target chronic inflammation and traditional CVD risk factors. Additionally, in PsA the management strategies should also focus on targeting metabolic components, including weight control (34).

## 3 Nonalcoholic fatty liver disease

Hepatic disease is one of the most important causes of morbidity and mortality worldwide. The liver is an organ susceptible to infections, autoimmune processes, and exposure to drugs or toxic compounds due to its large number of functions, including storage, metabolism, or detoxification of substances among others (35).

Different states can be recognized in the progression of liver dysfunction. The first sign of liver damage is steatosis. Histologically, NAFLD is defined by the presence of at least 5% hepatic steatosis and hepatocyte inflammation, and hypertrophy, regardless of the presence of fibrosis (36). Fat accumulation caused by IR represents the first hit of NAFLD. Thereafter, the fibrosis process can begin, in which the accumulation of fibrotic tissue replaces healthy tissue reducing liver function, called non-alcoholic steatohepatitis (NASH). The next stage is cirrhosis, a late phase of fibrosis where a high percentage of liver tissue is replaced by fibrotic tissue and liver functionality is substantially impaired. Lastly, the failure of the organ occurs when the function of the organ is found dramatically deteriorated leading to the development of hepatocarcinoma (37).

Some of the processes that are altered during this pathological sequence are increased lipogenesis, as well as insulin resistance, and the production of reactive oxygen species, which cause mitochondrial and plasma membrane damage. These two latest processes also promote macrophage infiltration which in turn increases the release of pro-inflammatory cytokines (38). In addition, metabolic pathways including amino acid metabolism, Krebs cycle, and transfer RNA biosynthesis, among others, are deregulated during the fibrosis process (39). Likewise, the development of extracellular matrix rich in type I and III collagens leads to the disruption of the organ structure (40).

About 25% of the world’s adult population is affected by NAFLD, due to unhealthy lifestyles, especially unhealthy diets and sedentary lifestyles (41). To describe the pathogenesis of NAFLD there are two theories, the “two-hit” theory proposed in 1998 and the “multiple-hit” postulated more recently in 2018 (42). The “first hit” is represented by the elevation in liver fat accumulation, followed by the “second hit” which includes inflammation, adipokines, mitochondrial dysfunction, and oxidative stress, processes needed for the progression from NAFLD to NASH and advanced fibrosis. Lately, the “multiple hit” add various processes, such as insulin resistance, lipotoxicity, inflammation, cytokines imbalance, innate immunity activation and microbiota, offering a more comprehensive description of the NAFLD pathogenesis.

## 4 Association of NAFLD and inflammatory arthritis

There is an increasing evidence suggesting an association between liver disease and IA. Although only a few studies have evaluated the mechanisms that are involved in the development of NAFLD/NASH in these diseases. Most of the studies carried out to investigate liver damage in patients with inflammatory arthritis have been mainly focused on the possible hepatotoxic effect of methotrexate, the first line treatment in RA and PsA (43). In addition, a recent systemic review indicated that the frequency of elevated liver transaminases during the first three years of treatment with low-dose of methotrexate in RA was 13 out of 100 patients/year, with a cumulative percentage of 31% (44). However, the mechanism of action underlying liver damage during methotrexate treatment is partially unknown, and it is not yet determined whether low doses can independently contribute to liver damage in these patients. Currently, the pathophysiological link between liver damage and inflammatory arthritis is unknown, although the potential mechanisms involved could be adipocytokines, altered lipid profile, obesity or the treatment administered.

Inflammatory diseases that share comorbidities with inflammatory arthritis, such as psoriasis, reefer NAFLD as a hepatic manifestation of MetS, and although its etiology is not entirely clear it has been postulated that inflammatory cytokines such as tumor necrosis factor-alpha (TNF-α), IL-6, IL1-β and resistin play a key role in the development of fatty liver disease (45).

Thus, the imbalance between lipid acquisition and elimination triggered by the inflammatory activity of IA could be linked to the development of NAFLD and NASH. Due to the limited number of studies adjusted for other non-alcoholic liver disease risks, such as MetS, it is challenging to predict whether a patient with RA or PsA may be predisposed to develop liver disease.

### 4.1 Liver damage in rheumatoid arthritis patients

As it was already mentioned, RA patients display metabolic alterations, such as dyslipidemia, increased body mass index, and type 2 diabetes among others, which have been shown closely related to the development of NAFLD (46). Thus, to determine the factors that are responsible for the liver damage in RA is a challenge, since it is difficult to conclude whether the liver damage can be considered an extra-articular manifestation due to the chronic inflammation and the metabolic comorbidities or it is resulting from hepatotoxicity due to the prolonged treatment (47).

The manifestations of liver abnormalities in RA have been reported by different studies for decades, mainly represented as unusual elevation of blood transaminases, increased levels of alkaline phosphatase and γ-glutamyltransferase (48). The percentage of RA patients with abnormal liver function varies depending on the study. Liver enzyme concentration abnormalities have been found in up 43% of RA patients (49), although these values can range in very wide ranges from 18% to 50% (47). A recent study performed on 2812 patients with RA with a mean follow up of 93.7 months, evaluated the influence of liver fibrosis burden in the mortality. The results showed that 3.2% out of the patients died, and this mortality rate was associated with age, sex (male), hypertension, T2DM, inflammatory markers and an index of liver fibrosis, which indicated the relationship between liver damage and mortality in RA patients (50).

Due to the heterogeneity of metabolic fatty liver diseases in 2020 a group of experts proposed a new nomenclature and diagnostic criteria from non-alcoholic fatty liver disease (NAFLD) to metabolic associated dysfunction disease (MAFLD) in order to more precise and inclusive diagnosis (51). A very recent study has analyzed the incidence of MAFLD in RA patients and its relationship with CVD risks. Using a Chinese cohort of 513 RA patients, the prevalence of MAFLD was about 20% and it was associated with an increase in CVD events (52).

Regarding the histopathological features, one study reported that the 65% of RA patients have pathological liver biopsies where at least 50% of patients displayed mild portal chronic inflammatory infiltrate and small points with necrosis, and 25% of patients had fatty liver (53).

Referring the mechanisms that are involved in hepatic damage in RA, inflammation seems to play a key role. Interleukin (IL)-20 is a protein implicated in inflammatory processes which are directly related to the pathogenesis of RA (54). Interestingly, Chiu and coworkers, evaluated the role of IL-20 in liver disease by several approaches, liver biopsies of 66 patients with several liver diseases compared to 3 healthy subjects, a mouse model with liver injury and *in vitro* experiments with a rat hepatocyte Clone-9 cells. They demonstrated that IL-20 was associated with liver damage, inducing the expression of TGF-β1 and P21 and inhibiting the hepatocyte growth due to the activation of hepatic stellate cells. Moreover, *in vivo* treatment with anti-IL-20 or anti-IL-20R1 in mice with liver injury, along with mice knockout for IL-20R1 showed protection against liver damage (35).

In this line, our research group has analyzed the potential impact of the RA on hepatic function by different strategies: a human cohort of 250 subjects, a mouse model with arthritis and *in vitro* studies with hepatocyte cell line (HepG2) treated with ACPAs. We identified that RA patients showed a subclinical liver alteration associated with inflammation, disease activity and levels of autoantibodies. Thus, we showed that enriched IgG-ACPAs isolated from RA patients profoundly impact hepatocytes promoting inflammation, oxidative stress and a defective glucose and lipid metabolism processes linked to liver injury. Besides, liver of mice with arthritis presented a chronic inflammatory state in parallel with an increase in the expression of macrophage markers, suggesting a potential liver damage induced by the arthritis (55).

### 4.2 Liver disease in PsA

Similar to RA patients, PsA patients have even an increase rate of disease-associated metabolic comorbidities including obesity, T2DM, dyslipidemia and hypertension which can directly be linked to the development of NAFLD (56). In the last ten years, the pathophysiological relationship between PsA and CVD comorbidities including liver disease has gained focus. Different studies have described that NAFLD occurs more frequently in PsA patients compared to the general population (56, 57). Through both, cross-sectional and longitudinal studies, these authors affirmed that the prevalence of the liver abnormalities in PsA is around 30% and is independently associated with BMI, MetS, disease activity and levels of CRP (57, 58). Other study described that about 65% of patients with PsA have NAFLD (59). In fact, when PsA and NAFLD co-exist the severity of both disease may increase significantly (60). For instance, the lipid profile was found more altered in patients with PsA-NAFLD compared to patients with PsA without NAFLD (61). Thus, PsA has become a risk factor for advanced liver fibrosis (60).

On the other hand, approximately 47% of patients with psoriasis suffer from NAFLD, while NASH can occur in one of five patients (45). In this sense, a recent meta-analysis by Bellinato and colleagues performed in more than 1.7 million individuals shows a significant association between psoriasis and a nearly two-fold increased likelihood of NAFLD compared to healthy controls. This risk was parallel to the severity of psoriasis (62). In PsA, NAFLD was significantly correlated with psoriasis lesions and disease severity index (PASI). When psoriasis is present, the likelihood of advanced NAFLD increases by approximately 60%, and progression to NASH is more likely (60).

Taking into account the relationship between psoriasis and PsA, a study conducted by Haque and coworkers demonstrated that there were not significant differences in the presence of liver fibrosis between patients with PsA and patients with psoriasis, although the latest group are metabolically more compromised (17). These results suggest that in the development of liver disease in PsA must be implicated other factors than the metabolic alterations, possibly intrinsic of the disease itself.

## 5 The interplay of cardiovascular disease and NAFLD

Liver diseases might affect cardiovascular functionality triggering the development of cirrhotic cardiomyopathy, hepatorenal syndrome, ascites, hepatopulmonary syndrome, portopulmonary hypertension, gastrointestinal bleeding, and hepatic encephalopathy and in turn, CVD can affect liver function and hepatic disease progression. Different mechanisms have been described as mediators of the relationship between the liver and systemic vasculature, such as inflammation, oxidative stress, endothelial dysfunction, and vasoactive mediator imbalance, among others (63).

Precisely, NAFLD has been associated with CVD, chronic kidney disease (CKD), and T2DM, deing CVD is the leading cause of death in patients with NAFLD (37). In fact, CVD risk factors such as ATH, dyslipidemia, obesity, and IR are usually accompanied by NAFLD (41). In addition, increasing evidence indicates that NAFLD is strongly correlated with an increased risk of any cardiovascular event independent of CVD risk factors such as cardiomyopathy, cardiac arrhythmias, and cardiac valvular calcification (64).

In the case of obesity, NAFLD constitutes an important factor in the course of the disease. It has been observed that NAFLD patients with obesity who reduce significantly their weight as part of their therapeutic treatment results in a regression of hepatic damage showed by a drop in serum levels of aminotransferases and triglycerides (65). However, NAFLD is not exclusively associated with the BMI, since its prevalence in patients with MetS and without obesity is alarmingly increased (66). In these cases where obesity is excluded, hypertriglyceridemia extensively contributes to the development of NAFLD (15). In fact, it is estimated that 5-8% of lean subjects (BMI<30) display NAFLD. In this sense, the results of a study by Feldman and colleagues point to a pronounced adipose tissue dysfunction in lean subjects with NAFLD and previously T2DM undiagnosed. These findings indicate the relationship between the alteration of adipose tissue and NAFLD, regardless of obesity (67).

It is noteworthy to mention the bilateral correspondence between NALFD and several components of MetS such as IR. On one hand, IR has been proposed to directly fuel NAFLD. Thus, adipose tissue-IR induces the release of free fatty acids that reach the liver causing a lipid overload in the hepatocytes, influencing the development of NAFLD. A great overview of the molecular mechanisms underlying NAFLD pathogenesis and IR has recently been published by Palma and coworkers (68). Secondly, a growing evidence postulate NAFLD as a key driver of IR. Under NAFLD conditions, the liver overproduces glucose affecting other tissues such as adipose tissue or skeletal muscle inducing a global state of insulin resistance (69).

Several studies highlight that NAFLD-associated MetS is a highly atherogenic condition. Patients with both conditions show an increase in the CIMT, as well as in the number of atherosclerotic plaques and plasma markers of endothelial dysfunction, thus increasing the prevalence of atherosclerosis.

The pathogenesis of cardiac dysfunction in NAFLD remains unclear, although it has been suggested that IR, dyslipidemia and the low-grade inflammatory state itself represented by liver fat may lead to lipid accumulation in the myocardium, epicardium, and pericardium. The necroinflammatory form of non-alcoholic hepatic steatosis might be implicated in cardiac dysfunctions through the release of different proinflammatory cytokines (C-reactive protein, IL-6, and TNF-α) with consequent cardiac alterations. Similarly, metabolically active epicardial adipose tissue produces a cascade of proatherogenic, proinflammatory, and prothrombotic adipocytokines leading to cardiovascular complications (70, 71).

Due to the close association between NAFLD and metabolic complications, the novel nomenclature for NAFLD, MAFLD, is intended to reflect the coexistence of different chronic liver diseases, resulting in a more accurate stratification of the pathogenesis of the disease, facilitating more effective treatment choices. Some studies have shown that the use of MAFLD criteria are more practical and effective than NAFLD in identifying patients at high risk of metabolic dysfunction and CVD (51, 72).

## 6 Assessment and monitoring of NAFLD/NASH

Different tools have been established for the diagnosis of liver disease in the clinical practice (**Table 1**). Most patients with NAFLD are usually asymptomatic, only some patients with NASH can display non-specific symptoms such as asthenia, malaise, and mild abdominal pain in the right hypochondrium. To make a differential diagnosis, a physical and anthropometric examination must be performed, in which habitual alcohol consumption must be excluded, as well as other causes of chronic liver disease. Following this, there are different types of diagnostic tools including classical blood biomarkers of the liver profile and biochemical indexes, imaging tests, and liver biopsy (73, 74).

**Table 1:** Diagnostic assessment of NAFLD/NASH.

Clinical examination	
	Physical examination, exclusion of alcohol consumption and other causes of chronic liver disease
**Serum markers and biochemical indexes**	
	Liver profile biomarkers
	Biomarkers of inflammation and fibrosis
**Imaging tests**	
	Abdominal ultrasonography
	Transient elastography (*Fibroscan^®^*)
	Controlled attenuation parameter (CAP™, *Fibroscan^®^*)
	Computed tomography (CT)
	Magnetic resonance imaging (MRI)
**Liver biopsy**	

Liver biopsy is the most effective means of assessing and classifying the degree of inflammation, the state of hepatocellular necrosis, and fibrosis, helping to determine the progression of NASH to a cirrhotic state. However, it is an invasive technique with potential complications and must be analyzed by a specialized hepatologist to avoid inter-and intra-observer errors. Therefore, this technique should not be used as a screening method for NAFLD even though its use in clinical practice is currently very common because no other method has demonstrated a complete correlation between clinical and analytical data and biopsy data (74, 75).

**Figure 1:**
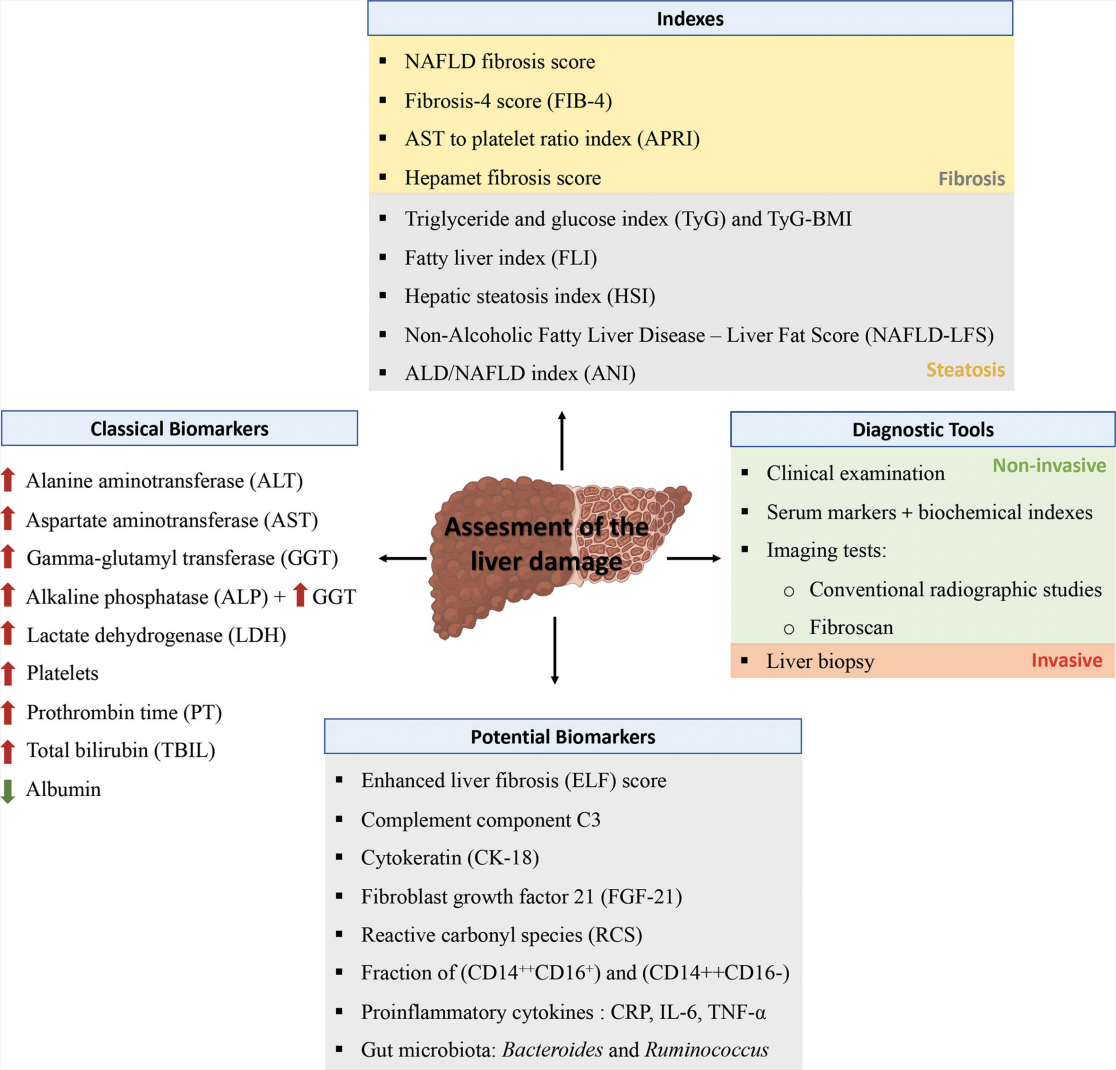
Assessment of liver disease. Different tools have been established for the diagnosis of liver disease in the clinical practice implying non-invasive (clinical examination and imaging test) and invasive techniques (liver biopsy). For screening method a number of scores based on analytical, clinical and anthropometric data are available to detect the risk of suffering liver fibrosis or steatosis. In this sense the alterations in various serum markers can evidence an alteration in the liver including liver enzymes, platelets, prothrombin, bilirubin or albumin. Finally, the latest studies point out to novel potential biomarkers that are associated with liver damage.

Although no biochemical marker has succeeded in displacing biopsy as the diagnostic standard for NAFLD/NASH, various serum markers and biochemical indexes together with different imaging tests are used for NAFLD screening (74, 76) (**Figure 1**).

### 6.1 Potential novel liver disease biomarkers

Recent studies have identified new molecules or factors related to liver disease in peripheral blood or microbiota that could be promising biomarkers to help in the diagnosis of this disease, although none of them are available for clinical use.

Thus, measurement of blood biomarkers of cell death is considered a non-invasive assessment of fibrosis in patients with chronic liver disease, as apoptosis is directly related to fibrosis. Cytokeratin (CK-18), the main intermediate filament protein of the liver, has been proposed as a biomarker of hepatocellular apoptosis. Immunoassays can detect CK-18 fragments after cleavage of cytokeratin by caspases during hepatocyte apoptosis, and this biomarker may discriminate between NAFLD and NASH (77). In addition, several population-based studies have demonstrated the presence of high levels of fibroblast growth factor 21 (FGF-21) in patients with NAFLD (78). Besides, one study reported that the combination of FGF-21 and CK-18 showed high accuracy as biomarkers in the detection of NASH (79).

As mentioned before, oxidative stress plays an important role in the development and progression of NAFLD. In fact, a study by Liu and coworkers, showed high levels of plasma reactive carbonyl species (RCS) in patients with NAFLD compared to healthy subjects, so authors claimed that increased RCS may considered as a direct risk factor for developing NAFLD (80).

Recently, an elevation of the total monocyte fraction in blood samples from NAFLD patients has been reported. As shown by Zhang et al., in a study on the influence of different monocyte subpopulations in NAFLD, they found that an elevated fraction of intermediate monocytes (CD14^++^CD16^+^) in peripheral blood, as well as a reduced fraction of classical monocytes (CD14^++^CD16^-^), was closely related to the development of liver disease (81).

Likewise, the presence of proinflammatory cytokines in the blood such as TNF-α, IL-6, and C-reactive protein (CRP) have been associated with the diagnosis of NAFLD (82). Thus, in different studies, it has been observed that TNF-α, CRP, and IL-6 values are significantly elevated in NAFLD patients compared to healthy controls (83, 84).

Lately, several studies have identified complement component 3 (C3) as a new biomarker for NAFLD. Complement C3 is an innate immune system protein synthesized mainly by hepatocytes, and its activation can lead to the appearance of large numbers of infiltrating neutrophils, as well as an abnormal increase in the expression of IL-8 and IL-6 in the liver tissue, ultimately leading to the pathogenesis of NAFLD (85). Thus, in 2013 a study conducted by *Wlazlo and coworkers* in over 500 individuals found that circulating levels of active C3 (C3a) were associated with hepatic steatosis and hepatocellular damage, this association was particularly prominent in alcohol-consuming individuals. Similarly, high plasma C3a levels were found to be related to hepatic fat content (86). Later, a cross-sectional study of more than 7000 individuals by *Xu and colleagues* analyzed the association between the presence of NAFLD and serum C3 levels. This study reported that patients with NAFLD had high serum C3 levels, regardless the presence of any metabolic syndrome component (87). However, another study conducted in a Chinese population of over 3000 participants with NAFLD found that the association between NAFLD and C3 levels was closely related to abdominal obesity, HOMA-IR, as well as other liver markers such as ALT, AST and GGT (88). In IA, Ursini and collaborators have shown that circulating levels of complement C3, in synergy with BMI, have a potential role as a possible biomarker of NAFLD in patients with RA (89). Studies should be done in PsA to evaluate the complement C3 as a biomarker of NAFLD in this disease.

On the other hand, the enhanced liver fibrosis (ELF) score, composed of tissue inhibitor of metalloproteinases 1 (TIMP-1), procollagen type III amino-terminal propeptide (PIIINP) and hyaluronic acid (HA), has been evaluated in different studies as a marker for different stages of fibrosis in liver disease. A study conducted in 2004 by *Leroy and colleagues* in 194 patients with chronic hepatitis C described the diagnostic utility of a panel composed of different circulating markers demonstrating that the combination of fibrogenesis and fibrinolysis markers, PIIINP and MMP-1, respectively, provides information on the fibrosis stage of patients (90, 91). Subsequently, numerous studies have validated this algorithm in cohorts of patients with chronic hepatitis C, demonstrating its diagnostic utility in both fibrosis and cirrhosis stages (92–94). Finally, a recent study by *Gawrieh and collaborators*, in a pediatric cohort of 166 children with NAFLD evaluated the diagnostic performance of the ELF score, which poorly discriminated between patients without fibrosis and those with mild fibrosis. However, it can be considered useful for discriminating children with advanced fibrosis (95). In IA patients, ELF score and procollagen-3 N-terminal peptide (P3NP) were elevated. The highest values were observed in RA patients, followed by psoriasis and PsA patients. Levels of the two test were increased in patients with mild-severe activity disease (96). The authors claimed that further research should be performed to validate ELF test in determining susceptibility for developing liver fibrosis in PsA and RA.

The human gut microbiota has been postulated as a new diagnostic biomarker of NAFLD progression to NASH. Several studies have associated various microbiota signatures with the severity of the liver disease. Increased Bacteroides in NAFLD patients compared to healthy donors, as well as elevated Ruminococcus in patients with stage 2-4 fibrosis compared to those patients without significant fibrosis have been shown [reviewed in (79)].

Finally, a recent study performed by Li and coworkers in 127 PsA patients (46 with NAFLD and 81 without NAFLD) has identified a prediction model for NAFLD consisting of the percentage of peripheral blood T helper 1 cells, the body mass index and the levels of triglycerides with a good efficacy and with a good clinical application value (61).

### 6.2 How can the liver disease risk be monitored in daily clinical practice?

Several indexes have been proposed to evaluate the risk of suffering NAFLD/NASH in asymptomatic subjects in the daily clinical practice. These tools are aimed to help the diagnose of liver disease, and screening between hepatic steatosis and fibrosis stage (summarized in **Table 2**).

**Table 2:** Indexes to determine hepatic steatosis or fibrosis and variables needed.

Screening tool	Variables	Reference
**Fatty Liver Index (FLI)**	BMI, waist circumference, triglycerides and GGT	(82)
**NAFLD fibrosis score**	Age, BMI, T2DM, ALT, AST, platelets and albumin	(83)
**Fibrosis 4 score (FIB-4)**	Age, ALT, AST and platelets	(84–86)
**Hepatic Steatosis index (HSI)**	BMI, gender, ALT, AST and T2DM	(47)
**AST to Platelet Ratio Index (APRI)**	AST, AST (upper limit of normal) and platelets	(50)
**Triglycerides and glucose index (TyG) and TyG-BMI**	Triglycerides and glucose and BMI	(87, 88)
**Hepamet fibrosis score**	Age, gender, T2DM, glucose, insulin, HOMA, AST, albumin and platelets	(89)
**Non-Alcoholic Fatty Liver Disease – Liver Fat Score (NAFLD-LFS)**	MetS, T2DM, insulin levels, ALT and AST	(90)
**ALD/NAFLD index (ANI)**	BMI, gender, mean corpuscular value, ALT and AST	(91)

BMI, body mass index; AST, aspartate aminotransferase; ALT, alanine aminotransferase; T2DM, type 2 diabetes mellitus; GGT, gamma glutamyltransferase; ALD, alcoholic liver disease; MetS, Metabolic Syndrome.

In 2006, *Bedogni and colleagues* created an algorithm based on BMI, waist circumference, triglycerides, and gamma-glutamyl transferase (GGT) for the detection of liver steatosis, with an good accuracy (AUC 0.84). This tool was named “fatty liver index” (FLI) (97). A negative likelihood of suffering fibrosis could be considered with values of FLI < 30, while a positive likelihood would be FLI values > 60.

Thereafter, *Angulo and coworkers* in 2007 validated a new non-invasive index called the NAFLD fibrosis score to screen NAFLD patients without fibrosis from those with advanced fibrosis. This index was able to predict advanced fibrosis in NAFLD patients with high accuracy by applying the high cut-off score (0.676). Thus, advanced fibrosis could be excluded when applying the low cutoff value (-1.455). With this score, liver biopsy could be avoided in 75% of the cases. This predictive model was presented as a clinically useful method using clinical and biochemical variables such as age, weight, and height, as well as the presence of T2DM/hypertension together with the aspartate aminotransferase/glutamic oxaloacetic transaminase (AST/GOT) ratio, alanine-aminotransferase/glutamic pyruvic transaminase ratio (ALT-GPT), platelet, and albumin values (98).

The FIB-4 index based on platelet count, AST, and ALT values together with age is considered of great value as a predictor of liver fibrosis. Thus, high levels of AST, ALT alongside with age and decreased platelet count correlate with increased liver fibrosis (99). To predict fibrosis in NAFLD, a cut-off of <1.30 has a predictive negative value to exclude advanced fibrosis of 90%, while a cut-off of >2.67 has a positive predictive value of 80% (100). FIB-4 has been widely considered to detect fibrosis in different scenarios and its accuracy is better than other non-invasive markers. FIB-4 could be used as a screening tool in the prevention of NAFLD in the high-risk population [reviewed in 101)]. Several studies have also tested FIB-4 as a marker for the diagnosis of liver disease in patients with RA, specially to monitoring the effect of methotrexate (50, 102, 103). These authors claimed that FIB-4 index can be a valuable marker to diagnose liver disease in RA patients under long-term methotrexate treatment and to stratify newly diagnosed patients under risk of premature death.

Lee and collaborators developed an index to detect the presence of NAFLD, “Hepatic steatosis index” (HSI), that comprises variables such as sex, ALT/AST ratio, BMI, and the presence of T2DM for its calculation. HSI can be used to select subjects with liver damage, so an HSI>36 indicates the possibility of having NAFLD and can be selected for ultrasonography and lifestyle modifications (104).

Furthermore, *the AST to platelet ratio index* APRI score is highly correlated with the fibrosis stage. It was initially developed for the estimation of liver fibrosis in patients with chronic hepatitis C (CHC) but was later validated in patients with NAFLD. The APRI index is based on platelet count and AST levels, as it is known that platelet count decreases and AST levels increase with the progression of liver fibrosis due to decreased thrombopoietin production by liver cells (105).

As mentioned, NAFLD is characterized by an excessive accumulation of triglycerides in the liver leading to hepatic insulin resistance, resulting in an overproduction of plasma glucose. A strong positive correlation has been observed between the triglyceride and glucose index (TyG) and the presence of NAFLD (99). In addition, the combination of TyG and BMI (TyG*BMI) has recently been shown to be more effective predictor of NAFLD than TyG alone in non-obese patients and patients with T2DM (106, 107).

Following this line, Ampuero and colleagues developed the Hepamet fibrosis score, a new risk scoring system for the development of advanced fibrosis in NAFLD patients. This index, validated in more than 2000 NAFLD patients using demographic data such as gender, age, and T2DM, anthropometric data such as HOMA, and biochemical values of glucose, insulin, AST, albumin, and platelets, effectively identified NAFLD patients with advanced fibrosis (108).

Similarly, Sandboge and colleagues studied the association between the presence of metabolic syndrome and T2DM in a pediatric population and the risk of developing NAFLD in the adulthood in a cohort of more than 1,000 subjects. Thus, dichotomous Non-Alcoholic Fatty Liver Disease - Liver Fat Score (NAFLD-LFS) score defined by the presence of variables such as metabolic syndrome, type 2 diabetes, and biochemical levels of insulin, AST and ALT may be associated with the risk of suffering NAFLD in adulthood (109).

Another study developed the ALD/NAFLD index to discriminate between patients with alcoholic liver disease (ALD) and patients with hepatic steatosis. Thus, this method combining the clinical variables of gender, BMI together with the biochemical values of mean corpuscular value (MCV), AST and ALT help to distinguish between ALD and NAFLD with high accuracy. In clinical practice, this index could be crucial in determining the treatment of hepatic steatosis as it allows prioritization of liver transplantation in those with a non-alcoholic basis. Also, if this index is combined with another variable, such as GGT, its differential diagnostic accuracy is more precise (110).

The practical advantages of all of these markers include their feasibility of measurement, their high applicability, their good inter-laboratory reproducibility and their accuracy in the screening for negative patients. Nevertheless, none of these scores are liver-specific and their values can be influenced by comorbidities, especially metabolic alterations, so the interpretation of the results should be cautious.

## 7 The liver and treatments in inflammatory arthritis

Treatment options to reduce inflammatory activity in IA begin with the use of non-steroidal anti-inflammatory drugs (NSAIDs) and corticosteroids. These drugs have been shown to produce a variety of side effects, most notably liver damage. Thus, the risk of liver damage is estimated to be ten times higher if NSAIDs are used in RA patients (111). In addition, conventional disease-modifying antirheumatic drugs (DMARDs) are often the first line of treatment, with methotrexate, being the main therapy for RA and PsA (110–112).

Over the last decade, numerous studies have been conducted on the possible secondary hepatotoxicity caused by methotrexate. The mechanism by which methotrexate causes liver damage is currently unclear, although it is thought to resemble non-alcoholic hepatic steatosis. Also, the hepatoxicity of methotrexate in rheumatic patients has been controversial due to different results obtained in the different studies.

A study conducted by Mori and colleagues on 800 RA patients treated with methotrexate suggested a strong association between low-dose methotrexate treatment and the development of NAFLD/NASH, highlighting the persistent transaminitis as the cause (113). Similarly, Bafna and colleagues observed an increase in liver stiffness by transient elastography (FibroScan^®^) after long-term (>3 years) treatment with methotrexate in RA patients, even when taking folic acid combined, which is postulated as a possible protective factor against methotrexate-induced liver injury (114).

However, a more recent study in RA and PsA patients treated with methotrexate at weekly doses of less than 25mg in association with folic acid showed no liver toxicity (102). These results are in agreement with those obtained by Darabian and collaborators who evaluated the association between methotrexate treatment in IA patients and liver damage, thus they conclude that there is no significant correlation between cumulative methotrexate dose and liver stiffness, even at high methotrexate doses (115).

On the other hand, patients with psoriasis may be more susceptible to methotrexate-induced hepatotoxicity than those with RA, so a current population-based study by Gelfand and coworkers compared the risk of liver injury among patients with psoriasis, PsA or RA treated with methotrexate for more than 15 years. Their results show that patients with psoriatic disease treated with methotrexate are more likely to suffer liver complications than RA patients. However, the cause of liver disease as a result of methotrexate use in these diseases cannot be clearly determined, especially in severe cases such as cirrhosis (116).

Liver complications associated with psoriasis may be due to the ‘dermal axis’ in which lymphocytes and keratinocytes produced by psoriasis lead to an increase in proinflammatory cytokines that are directed towards the liver promoting metabolic alterations until eventually occurs the development of NAFLD (117). However, Gay SY in a letter to the editor regarding the latter study advocated differentiating between the notion of ‘increased methotrexate hepatotoxicity’ and ‘more severe liver disease’, thus recommending to focus future studies on mild to moderate liver disease, as the risk of cirrhosis may not be attributed to methotrexate hepatotoxicity as previous studies have already shown (117). In short, a misinterpretation of the origin of liver damage in IA could lead to the discontinuation or definitive suspension of methotrexate, an effective drug in the treatment of these diseases.

A cross-sectional study performed by our group on RA patients showed a subclinical alteration of liver enzymes associated with inflammation and autoimmunity, suggesting that RA could be associated with liver abnormalities induced, at least partially, by the effect of ACPAs. Similarly, in a mouse model of collagen-induced arthritis (CIA) in obese mice treated with methotrexate or leflunomide, we observed that methotrexate could affect liver function in the presence of pre-existing subclinical liver impairment such as obesity (118).

Leflunomide is another DMARD widely used in the treatment of RA and PsA. This drug may induce potential deleterious effects on the liver through different molecular mechanisms such as the induction of mitochondrial and endoplasmic reticulum stress or alterations in metabolic and inflammatory pathways promoting hepatic fibrosis [reviewed in (119)]. In fact, the use of leflunomide at higher doses of 20 mg/day might be associated with a higher incidence of liver damage. In a study carried out on RA patients, leflunomide increased liver enzymes 2-3 times, these levels were normalized after 4-6 weeks of withdrawal (120). In addition, the combination of leflunomide with methotrexate has been shown to induce a greater degree of liver fibrosis in animal studies (121). In this sense, this combination is contraindicated in patients with liver alteration. Thus, the use of leflunomide should be considered cautiously, monitoring liver transaminases throughout the treatment regimen.

On the other hand, treatment with biologic DMARDs such as TNF-α and IL-6 signaling blockers has had a major impact on the treatment of these inflammatory diseases. Liver damage caused by anti-TNFα is rare. In some cases, a mild increase in aminotransferases can occur, up to more severe forms. However, the hepatotoxic mechanism associated with these drugs remains to be clarified (74). In contrast, potentially useful benefits of anti-TNF-α treatments in NAFLD have been proposed. In a murine model, anti-TNF-α antibodies were shown to be effective in reducing liver inflammation, necrosis, and fibrosis (122).

Regarding the inhibitors of IL-6 signaling used for the treatment of RA, such as tocilizumab and sirukumab, the generation of serious liver abnormalities is also scarce under this treatment regimen. The most common effect in the liver of these anti-IL-6 is the elevation of transaminases (AST and ALT), but in most cases, this increase mainly occurs in the first year of treatment and is reverted after discontinuation (123, 124). It seems that the negative effect of anti-IL-6 in the liver is due to the role of this interleukin in protecting against hepatic damage and participating in the regeneration of the liver (125). Thus, treatment with the inhibitors of IL-6 signaling plus DMARDs such as methotrexate would block the recovery from liver damage caused by this latter.

## 8 Conclusions

- There is a close interplay of cardiovascular disease (CVD) and hepatic damage, where adipose tissue dysfunction associated with metabolic alterations such as obesity, hypertriglyceridemia, insulin resistance or chronic inflammation is directly involved.
- In rheumatoid arthritis and psoriatic arthritis, in which the CVD risk and metabolic comorbidities are high, hepatic alterations are more prevalent. The postulated factors implicated in liver damage are chronic inflammation, metabolic disorders, and treatments administered.
- Several noninvasive scores to monitor the risk of liver disease have been developed, most of them taking into account the increase of BMI as the principal inductor of hepatic damage. Further biomarkers need to be researched to correctly identify the liver abnormality in non-obese patients.
- The liver abnormalities in inflammatory arthritis usually appear clinically silent, accompanied by the alteration in the levels of transaminases which can lead to the development of NAFLD. In this sense, rheumatologists should monitor regularly the risk of hepatic disease through the use of noninvasive tools (hepatic indexes) independently of obesity or the therapeutic regimen.
- Physicians should be cautious about prescribing methotrexate, leflunomide, and NSAIDs to patients having advanced liver disease. In addition, the combination of these drugs that increase the burden on the liver should also be avoided in patients with obesity or metabolic syndrome.

## Author contributions

All authors listed have made a substantial, direct, and intellectual contribution to the work and approved it for publication.

## Funding

The authors’ work is supported by grants from the Instituto de Salud Carlos III (PI20/00079, PI21/005991, and RICOR-RD21/0002/0033), and the Andalusian government (1381035-F) co-financed by the European Regional Development Fund (ERDF), a way to make Europe, Spain, MINECO (RyC- 2017-23437, the Andalusian Foundation of Rheumatology (FAR) and Consejería de Conocimiento, Investigación y Universidad, Junta de Andalucía (P20_01367). CL‐P was supported by a contract from the Junta de Andalucia (Nicolas Monardes program).

## Conflict of interest

The authors declare that the research was conducted in the absence of any commercial or financial relationships that could be construed as a potential conflict of interest.

## Publisher’s note

All claims expressed in this article are solely those of the authors and do not necessarily represent those of their affiliated organizations, or those of the publisher, the editors and the reviewers. Any product that may be evaluated in this article, or claim that may be made by its manufacturer, is not guaranteed or endorsed by the publisher.
